# Impact of Collaborative Nursing Care on Health Outcomes of Mental Health Day Hospital Users: A Mixed Methods Study

**DOI:** 10.1111/jpm.13133

**Published:** 2024-11-11

**Authors:** Ana Ventosa‐Ruiz, Antonio R. Moreno‐Poyato, Cristina Cañete‐Massé, Júlia Rolduà‐Ros, Isabel Feria‐Raposo, Karina Campoverde, Montserrat Puig Llobet

**Affiliations:** ^1^ Departament d'Infermeria de Salut Pública, Salut Mental i Maternoinfantil, Facultat d'Infermeria Universitat de Barcelona Barcelona Spain; ^2^ Grup de Recerca en Cures Infermeres de Salut Mental Psicosocials i Complexitat de la Universitat de Barcelona (2021 SGR 1083) L'Hospitalet de Llobregat Spain; ^3^ Hospital de día de l'Hospitalet de Llobregat Benito Menni CASM Barcelona Spain; ^4^ Departament de Psicologia Social i Psicologia Quantitativa Universitat de Barcelona Barcelona Spain; ^5^ Parc Sanitari Sant Joan de Deu Sant Boi de Llobregat Spain; ^6^ FIDMAG Germanes Hospitalaries Research Foundation Barcelona Spain; ^7^ CIBERSAM Institute of Health Carlos III Madrid Spain; ^8^ Centre Psicoterapia Barcelona‐Serveis Salut Mental (CPB‐SSM) Clínica Llúria Barcelona Spain

**Keywords:** collaborative nursing care, mental health recovery, mixed methods, participatory action research, positive mental health, therapeutic relationship

## Abstract

**Introduction:**

Given that recovery‐oriented care focuses on empowering individuals with mental health challenges, collaborative care can be an effective tool for nurses in mental health day hospitals.

**Aim:**

To deepen knowledge about the impact of collaborative nursing care for improving health outcomes of mental health day hospital users.

**Methods:**

A sequential and transformative mixed methods study was designed. In the first phase of this mixed methods study, quantitative data were collected from 144 users of mental health day hospitals. In phase two, a group of users underwent an intervention based on collaborative nursing care, through participatory action research, and qualitative data were collected. Finally, quantitative data were again collected from all users.

**Results:**

The users who participated in the intervention group improved quantitatively in terms of the quality of the therapeutic relationship. They also improved at different stages of the recovery process, thus the qualitative results confirmed that collaborative nursing care was an essential component in their recovery process.

**Discussion:**

The findings highlight the critical role of collaborative nursing care in health outcomes. The therapeutic relationship was identified as a key factor in facilitating patient empowerment.

**Implications:**

The study supports implementing collaborative nursing care models in mental health settings to enhance patient outcomes.

**Trial Registration:**

ClinicalTrials.gov identifier: NCT04814576

**Summary**

- What is known on the subject:
  ○ Recovery‐oriented care focuses on empowering individuals with mental health challenges, emphasising their strengths, autonomy and personal goals.
  ○ Positive Mental Health is linked to a person's ability to manage stress, maintain meaningful relationships and contribute to their community.
  ○ Collaborative healthcare involves active collaboration between mental health nurses and patients, with the aim of improving health outcomes by involving patients in decision‐making.
- Originality:
  ○ The study explores how collaborative nursing care within mental health day hospitals can enhance the therapeutic relationship and support the recovery process.
  ○ It demonstrates that collaborative nursing care improves not only the practical aspects of recovery, but also has a positive impact on positive mental health, offering a holistic approach to mental health nursing care.
  ○ The research provides evidence on how collaborative nursing care practices can lead to better engagement, increased feelings of belonging and more supportive networks for patients.
- Significance:
  ○ The findings suggest that implementing collaborative nursing care models in mental health settings can significantly improve patient outcomes, particularly in recovery and positive mental health.
  ○ Mental health services could benefit from adopting collaborative nursing care approaches to foster stronger therapeutic relationships and enhance overall well‐being for service users.
  ○ These insights can guide policy changes and the development of more person‐centred care practices in mental health services.

## 1 Introduction

In recent decades, the paradigm of mental health care has evolved significantly from an approach focused exclusively on symptom control and disorder management to a perspective that values and promotes the recovery of the whole person. The concept of “recovery process” in mental health has gained recognition worldwide as a philosophy of care that emphasises hope, autonomy and the ability of individuals to overcome the challenges associated with mental disorders (Davidson et al. 2021; Deegan 1988; Ellison et al. 2018).

The recovery process is based on the belief that, regardless of diagnosis, each individual has the potential to move toward a meaningful and purposeful life. It involves a holistic understanding of the person and their context, considering not only clinical aspects, but also the social, cultural and personal factors that influence the individual's well‐being (Roberts and Boardman 2013). This approach, supported by empirical evidence, recognises that recovery is not a final destination, but rather a unique and continuous journey for each individual (Dell, Long, and Mancini 2021; Leamy et al. 2011; Whitley, Palmer, and Gunn 2015).

Health policies have also evolved to align with this person‐centred approach (World Health Organization 2022). Increasingly, mental health systems worldwide are adopting strategies and programs that seek to empower individuals in their own recovery process and encourage active participation in decisions about their care (Corrigan et al. 2019). Collaborative care, based on shared decision‐making, can be framed within these strategies (Metz et al. 2019; Verwijmeren and Grootens 2024), advocating the promotion of self‐determination, respect for cultural diversity and collaboration between health professionals and service users. This way of delivering care through empowerment has increasingly demonstrated its effectiveness (Castro et al. 2016).

The relationship between the concept of recovery and positive mental health (PMH) is close and significant. PMH is defined as a state of well‐being in which the individual is aware of their own capabilities, can cope with the normal stresses of life, work productively and fruitfully, and is able to make a contribution to their community. This concept is not limited to the absence of mental disorders. It includes a number of factors that contribute to the optimal development of the individual and their positive adaptation to life circumstances (Ovidio Muñoz et al. 2016). Both concepts, recovery and PMH, complement and reinforce each other, and together, play a crucial role in the well‐being and improved quality of life of people facing mental health challenges. Both focus on well‐being and improving people's quality of life, as well as seeking to improve people's overall experience and help them thrive in their daily lives, by emphasising the active role of the individual in their own well‐being (Iasiello et al. 2019). This model was developed in 1958 by Jahoda (1959) under the title “Current concepts of positive mental health,” where the concept is articulated in six interrelated general criteria: attitudes toward the self, growth, development and self‐actualisation, integration, autonomy, perception of reality and environmental mastery. Later, Lluch Canut (1999), based on the results of different analyses of Jahoda's original model, proposed a new model of PMH, based on six explanatory factors of PMH: personal satisfaction, prosocial attitude, self‐control, autonomy, problem‐solving and self‐actualisation and interpersonal relationship skills. To evaluate this model, the author created the Positive Mental Health Questionnaire (PMHQ).

In parallel, the relationship between mental health recovery and the therapeutic relationship (TR) is fundamental and reciprocal in the process of care and treatment for people facing mental health challenges. The TR promotes person‐centred care and shared decision‐making, which are essential elements of the mental health recovery process (Davidson et al. 2021). By actively involving the patient in their own care, autonomy and empowerment are fostered, which are fundamental to recovery and PMH (Corrigan et al. 2019). The nurse not only acts as a care provider, but also as a facilitator of the patient's holistic well‐being, helping them to develop coping strategies and skills that enable them to better manage their condition and lead a meaningful and productive life. The TR provides a safe and trusting environment where people can express their thoughts and emotions without fear of judgement (Felton, Repper, and Avis 2018). A strong TR promotes collaboration and active participation of the patient in their own recovery process (Hamovitch, Choy‐Brown, and Stanhope 2018), which is essential to empower the individual and promote a sense of control over their own life. An empathetic, understanding and supportive TR can make a significant difference on the road to recovery and the achievement of an emotionally satisfying and meaningful life.

This study is justified by the need to deepen the understanding of how collaborative nursing care can significantly improve the recovery process in mental health, the TR and PMH. As health policies and models of care evolve toward more person‐centred approaches, it is crucial to have empirical evidence to support these practices (World Health Organization 2022; Corrigan et al. 2019). Collaborative care, founded on shared decision‐making and patient empowerment, not only has the potential to improve clinical outcomes, but also to enrich the overall experience of individuals on their road to recovery (Metz et al. 2019; Verwijmeren and Grootens 2024). In addition, the integration of this care with the PMH factors proposed by Lluch can offer a more complete and holistic view of well‐being, facilitating more effective and sustainable interventions (Lluch Canut 1999). Therefore, this study not only seeks to provide valuable data for clinical practice and health policies, but also to promote a cultural change in mental health care that emphasises collaboration, autonomy and personal growth.

Consequently, the objectives of this study were to explore the process of change taking place in the recovery process of mental health day hospital users who received the collaborative nursing care intervention and to evaluate the impact of the collaborative care intervention in terms of the changes produced in the stage of the recovery process, the level of PMH and the quality of the TR with the nurse.

## 2 Methods

### 2.1 Design

To meet the main objectives, a sequential and transformative mixed methods study was designed (Teddlie and Tashakkori 2012) involving three phases. In phases one and three, quantitative data were collected through a non‐randomised, two‐arm, parallel design. In phase two, the intervention was carried out, providing collaborative nursing care through the co‐design and implementation of group activities using qualitative methodology based on participatory action research (PAR). Given the sequential mixed methods approach underpinning the design, the integration of the data was carried out at both the design and result interpretation stages. During the design phase, we ensured that the data collection methods were robust and comprehensive, allowing for a seamless integration of diverse data sources. Furthermore, we adopted a contiguous approach to integration, presenting our findings within a single report but in separate sections for qualitative and quantitative results. This method allowed us to maintain the integrity of each data type while providing a comprehensive view of the research outcomes (Teddlie and Tashakkori 2012). The COREQ‐32 and CONSORT guidelines were followed to conduct this study.

### 2.2 Study Setting and Participants

The study setting was three adult mental health day hospitals in the metropolitan area of Barcelona (Spain). The three centres belong to the same institution and are part of the public mental health network of Catalonia. These centres share the same management, and therefore, the same care program is in place. Additionally, these 3 days hospitals are located in neighbouring communities, resulting in a population with similar characteristics. The allocation of the hospitals in the control and intervention groups was determined based on attendance criteria. The two smallest hospitals were selected for the control group, while the hospital with the highest attendance was chosen for the intervention group. The study population consisted of the users requiring treatment in the mental health day hospitals included in the study. The day hospital attends people over 18 years of age, with no maximum age limit, who are in a situation of decompensation of their basic pathology, who do not require admission to an acute unit. They receive care 5 days a week, where they are attended by a multidisciplinary team.

### 2.3 Participant Selection and Recruitment Criteria

The study inclusion criteria for participating users were as follows:

A Users of the selected day hospitals over the age of 18 years old.

B Acceptance of the terms of the study and informed consent.

The exclusion criteria for participating users were:

A Hospital admission for a period of < 1 week.

B Physical or psychological conditions that hinder the application of collaborative nursing care through the co‐design of group activities.

All persons who met the inclusion and exclusion criteria and who agreed to participate in the study were invited. No user refused to participate in the study. Participants were incorporated consecutively as they were admitted to the three units that were involved in the study.

**FIGURE 1:**
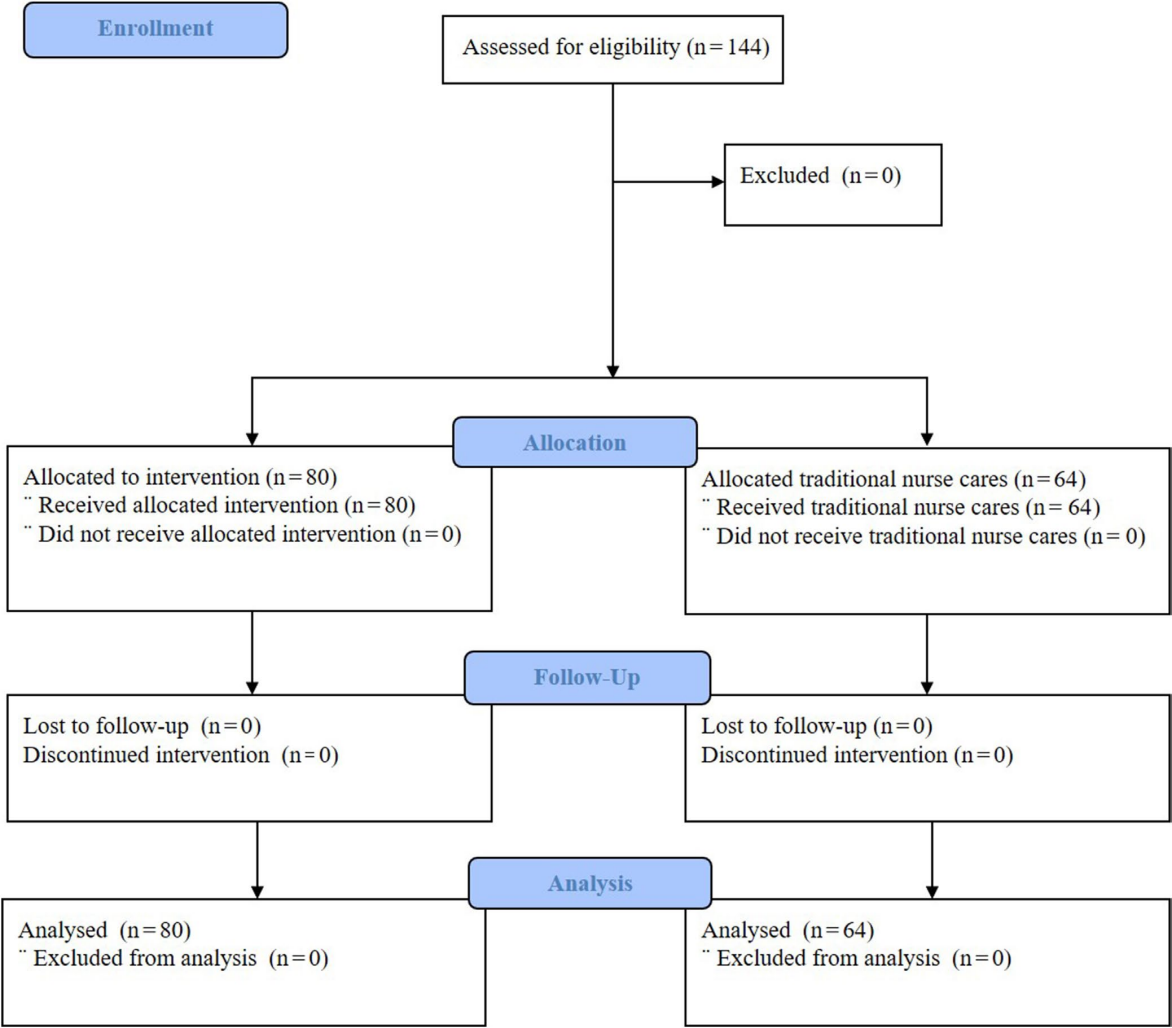
CONSORT Flow diagram.

Users admitted to the units between January 2021 and December 2022 were invited to participate. Assignment to either the control or intervention group depended on the individual's place of admission (Figure 1).

## 3 Phases I and III: Quantitative Inquiry

### 3.1 Outcome Measures

#### 3.1.1 Primary Result

Changes in the users' recovery process were evaluated with the Stages of Recovery Instrument (STORI) (Andresen, Caputi, and Oades 2006). This is a self‐report questionnaire of 50 items grouped into 5 dimensions of 10 items. Each dimension is related to one of the recovery processes (moratorium, sensitisation, preparation, reconstruction and growth). The items are scored from 0 “not true at all at this time” to 5 “totally true at this time”, resulting in a score for each stage, ranging from 0 to 50. The participant is assigned to the stage with the highest score. The questionnaire has been validated in the Spanish population, obtaining a Cronbach's alpha of 0.86 (Lemos‐Giráldez et al. 2015) and therefore presents adequate psychometric properties.

#### 3.1.2 Secondary Results

The quality of the TR between nurses and users was assessed using the Working Alliance Inventory‐Short (WAI‐S) scale. The short version of this scale contains 12 items, and each item is rated on a scale ranging from 1 (never) to 7 (always).

The overall WAI‐S score range is 12–84 points. The higher the score, the higher the TR. This questionnaire has three dimensions: (i) bonds: the bond between the patient and the nurse; (ii) goals: the agreement between the patient and the nurse on the goals of therapy and (iii) tasks or activities: the agreement between patient and nurse on the tasks or activities to be performed. The Spanish version of the WAI‐S has good reliability and validity, with a Cronbach's alpha of 0.93 (Andrade‐González and Fernández‐Liria 2016).

The level of PMH was assessed using the positive mental health (PMH) scale, developed and validated by Lluch Canut (1999) which studies the level of PMH, a concept previously developed by Jahoda (1959). The scale consists of 39 scorable items, for which the lowest value is “always or almost always,” and the highest value is “never or almost never.” The items belong to six dimensions (personal satisfaction, prosocial attitude, self‐control, autonomy, problem‐solving and self‐fulfilment and interpersonal relationship skills). The questionnaire has shown adequate internal consistency values in different populations with Cronbach's alpha values of 0.89 (Roldán‐Merino et al. 2017) and therefore adequate psychometric properties.

### 3.2 Quantitative Data Collection Procedure

Users who agreed to participate in the study and signed the informed consent, both in the case of the intervention and control groups, were given a form containing a questionnaire with sociodemographic and clinical data and the three evaluation instruments by a member of the research team not directly involved in the care of the users. Participants did not receive any information about their inclusion in the intervention or control group. Each user was assigned a participant code to ensure the anonymity of the participating users. When the user was discharged, a person from the research team collected follow‐up data in the same manner using a new form.

### 3.3 Quantitative Data Analysis

The quantitative analysis focused on the numerical differences obtained through the STORI, WAI‐S and PMH questionnaires before and after the intervention, and their comparison with the subjects in the control group. Descriptive statistics were used to describe the characteristics of the participants, and the scores obtained on the scales, using the arithmetic mean and standard deviation for quantitative variables and the frequency and percentage for qualitative variables. The differences between the baseline scores obtained and the follow‐up assessment were estimated by applying parametric tests (Student's *t*‐test for independent groups) in the case of quantitative variables, first the assumptions of normality and homogeneity of their variances were verified. When the opposite was true, nonparametric tests were used, using the Mann–Whitney *U* test for quantitative variables. In the case of qualitative variables, the Chi‐square test was used if the application conditions were met (expected frequencies greater than five) or Fisher's exact test if they were not. Finally, to analyse whether there were statistically significant differences before and after the intervention, taking into account the group (control or treatment), mixed ANOVA models were used. A significance level of *p* < 0.05 was considered and the effect size was estimated for each of the analyses.

## 4 Phase II—Qualitative Inquiry

### 4.1 Collaborative Nursing Care Intervention

#### 4.1.1 Data Collection Procedure

The intervention was carried out using the participatory action research methodology developed through a cycle of four well‐defined steps (Susman and Evered 1978) that were continuously repeated throughout the process. PAR is an appropriate methodology for the implementation and evaluation of changes, since it enables the generation of knowledge in a democratic, cooperative, transparent and effective manner, as well as intervening in the changes in people's daily lives (Moreno‐Poyato et al. 2023).

For each user who joined the study, at least one complete cycle of PAR was performed before the user was discharged. The beginning of the cycle began with the diagnostic stage, which was performed at the time of admission to the day hospital. The nurse, through an individual semi‐structured interview, explored the user's meaning of the concept of recovery along with the individual perception of the facilitating and limiting elements of this process. Subsequently, in the second or planning stage, through a focus group with other users, the meaning of recovery was collectively discussed, as well as its limitations and facilitators, where individual or collective action plans were shared among users to work and improve their level of recovery in stage three or the action stage. Once the action plan agreed upon by the users and the nurse had been carried out, the cycle concluded with the fourth stage of evaluation, where an individual action was again carried out in the form of a semi‐structured interview to evaluate the recovery process.

### 4.2 Qualitative Data Collection Techniques

#### 4.2.1 Semi‐Structured Interview

The conversations were recorded with the prior consent of the participant and subsequently a written transcript was produced, which required validation by the user to avoid possible biases. The interviews were conducted in a room, without interruptions and lasted an average duration of 40 min (Min. 20 Max 57). The user was given the possibility of ending the interview at any time they wished without explanation. A script was used to conduct the interview.

#### 4.2.2 Focus Groups

Focus groups were recorded. A weekly group was held at the day hospital facilities, during the operating hours of the day hospital, with an average duration of 45 min (Min 38 Max 48). The user was given the possibility of leaving the group if desired. A support script was followed during the focus groups. The researcher who conducted the focus groups was aware that her words and attitudes could influence group dynamics; therefore, she took measures to minimise this potential bias.

#### 4.2.3 Researcher's Diary

As a tool for reflection and in order to follow up the research process, a field diary prepared by the principal investigator was kept. This diary was kept by the unit's referring nurse, who was the principal investigator of the present study.

### 4.3 Qualitative Data Analysis

The qualitative content analysis method was used (Crowe, Inder, and Porter 2015). The data obtained from both the focus groups and the interviews were transcribed verbatim. Then, once the authenticity of the transcripts had been verified by the participants, the text was broken down into descriptive codes assigned according to their purely semantic content. In the second stage, these codes were grouped into more analytical subcategories, so that the initial codes were grouped according to the meaning of the linguistic units and their combinations. This resulted in a third hierarchical stage, where, considering the semantic analysis of the previous subcategories, they were categorised according to the objectives of the study. It should be noted that the data analysis was carried out by two researchers, jointly in the first stage of data coding and independently during the subsequent analytical process and again together in the comparison of their results. The analysis of these data was performed using the QRS Nvivo v12 program.

### 4.4 Ethical Considerations

Authorisation was obtained from the centre's management and project approval was granted by the Ethics Committee of the institution where the study was conducted, FIDMAG Hermanas Hospitalarias (PR‐2020‐10). All participants were able to voluntarily withdraw from the study at any time. For this reason, users were given a sheet with all the precise information about the study to be carried out and their written consent was requested.

## 5 Results

A total of 144 users from three adult mental health day hospitals participated in the study; none withdrew during the data collection process. The distribution was 64 users in the control group and 80 in the intervention group. Table 1 Shows the sociodemographic characteristics of both groups with their corresponding statistical tests. There were no statistically significant differences between the control group and the intervention group in age or sex. The results show some initial differences between the intervention group and the control group, basically at the level of the scores in the Rebuilding and Growth recovery stages, as well as the perception of the TR with the reference nurse, with better scores in the control group than in the intervention group. Therefore, initially, the users who were included in the control group scored higher on the quality of their relationship with the nurse and the Rebuilding and Growth stages of the recovery process.

**TABLE 1:** Differences in socio demographic characteristics and outcomes of participants at baseline.

Variable	Control *n* = 64	Intervention *n* = 80	*T* student
Age	42.05 (13.60)	44.71 (15.19)	*T* = 1.095
			*p* = 0.25
			df = 142
Gender (women)	51.56%	53.76%	χ^2^ = 0.068
			*p* = 0.794
			df = 1
Moratorium	26.453 (12.036)	26.725 (12.211)	*T* = 0.134
			*p* = 0.894
			df = 142
Awareness	26.725 (12.211)	25.863 (10.366)	*T* = 1.660
			*p* = 0.099
			df = 142
Preparation	28.750 (10.374)	23.175 (11.500)	*T* = 1.964
			*p* = 0.051
			df = 142
Rebuilding	26.813 (10.441)	22.250 (12.075)	*T* = 2.389
			*p* = 0.018
			df = 142
Growth	22.172 (11.020)	18.250 (11.491)	*T* = 2.072
			*p* = 0.040
			df = 142
PMH 1 Personal satisfaction	18.875 (6.477)	17.575 (7.277)	*T* = 1.118 *p* = 0.370 df = 142
PMH 2 Prosocial attitude	15.969 (2.624)	14.488 (3.673)	*T* = 3158.500 *p* = 0.007*
PMH 3 Self‐control	11.531 (3.563)	10.387 (3.733)	*T* = 1.864 *p* = 0.881 df = 142
PMH 4 Autonomy	13.391 (3.407)	12.963 (3.644)	df = 142
PMH 5 Problem‐solving and self‐actualisation	23.813 (5.603)	21.863 (4.414)	*T* = 2.336 *p* = 0.056 df = 142
PMH 6 Interpersonal relationship skills	17.906 (4.569)	18.038 (4.465)	*T* = −0.173 *p* = 0.659 df = 142
PMH total	101.484 (17.064)	95.313 (20.155)	1.953
			*p* = 0.055
			df = 142
WAI‐S	67.016 (10.293)	57.112 (13.260)	*T* = 4.907
			*p* = < 0.001**
			df = 142
Goals	21.406 (3.923)	19.175 (4.269)	*T* = 3.230
			*p* = 0.002
			df = 142
Bonds	23.328 (4.543)	18.700 (5.408)	*T* = 5.473
			*p* = < 0.001**
			df = 142
Tasks	22.281 (4.177)	19.238 (5.077)	T = 3.862
			*p* = < 0.001**
			df = 142

Abbreviations: PMH, positive mental health; WAI‐S, working alliance inventory‐short.

* Does not meet homocedasity. therefore we interpret Mann–Whitney.

** Brown‐Forsythe test is significant (*p* < 0.05). Suggesting a violation of the equal variance assumption.

### 5.1 Stage 1‐Diagnosis

In this first stage, a semi‐structured individual interview was conducted with the patients who took part in the intervention to explore the meaning that the person interviewed gave to the recovery process.

The data revealed that participants experienced a profound perspective on the recovery process. Users highlighted the challenging nature of this journey, characterised by its complexity and demands. They felt that the attitude with which they approached this process was fundamental, and a deep inner desire to overcome and improve was key. Beyond simply facing the challenges, participants emphasised the importance of adopting a resilient and proactive mindset. Emphasis was placed on the fact that the road to recovery involved not only facing adversity, but also actively embracing the drive to change and move toward personal improvement.

**FIGURE 2:**
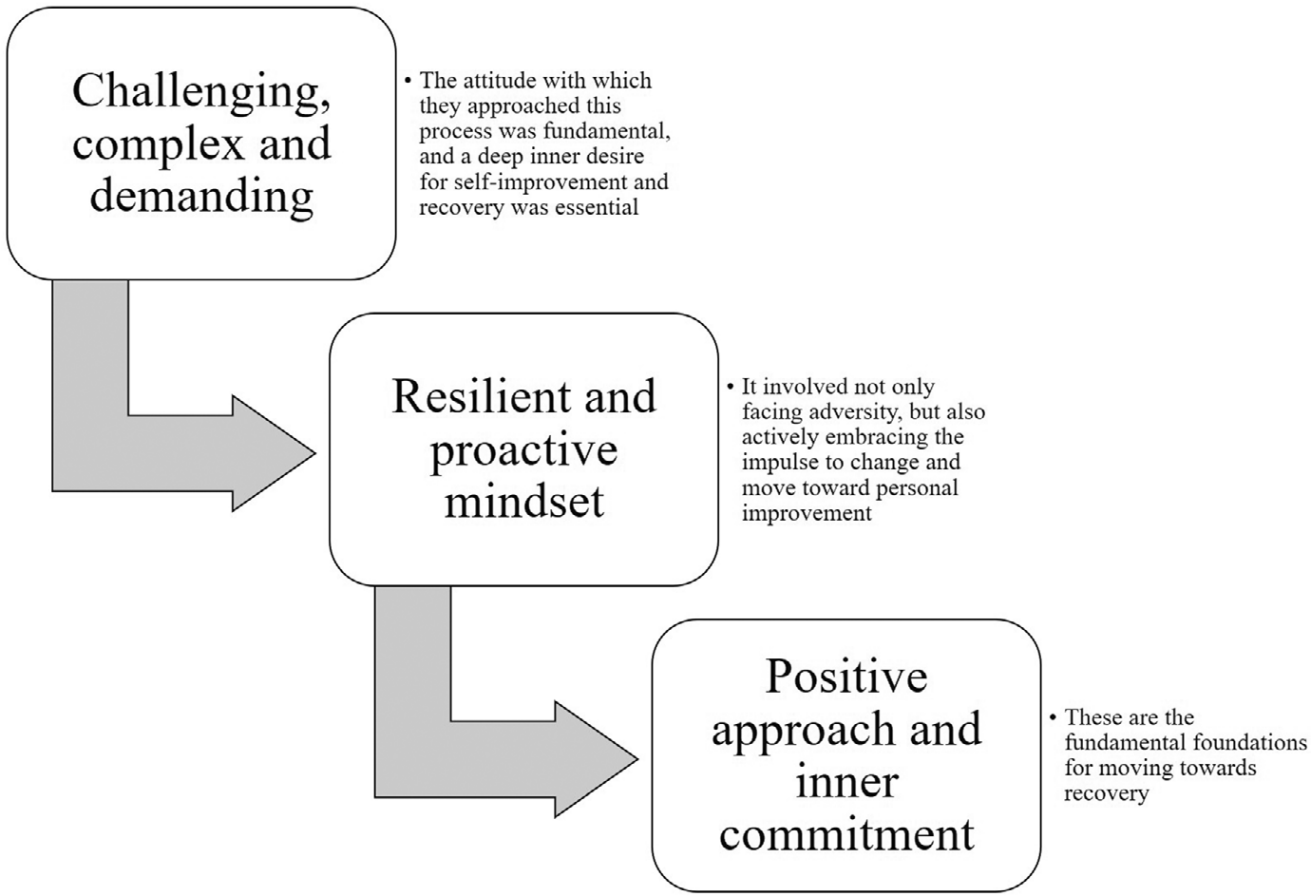
Meaning of the recovery process for participants.

People's willingness to face obstacles, combined with a genuine yearning to make progress, emerged as a crucial factor in the recovery process. Participants emphasised the need for a positive approach and inner commitment as a fundamental foundation for moving toward recovery (Figure 2).

if you don't have the will to improve, you won't be able to get out of the ditch you are in. (11)

Different approaches to recovery emerged in the interviews. While some users stated that recovery involved accepting and learning to live with their mental health conditions without hiding them, others expressed the hope of overcoming their problems altogether. These diverse perspectives underscored the complexity and uniqueness of the pathways to mental recovery, highlighting the importance of understanding and respecting different individual visions and goals in this process.

Look, I hide from my friends when I see them because I have never explained it to them in all the years that I've been like this, I've never talked about it with them, with any of them, and I hide from my family so as not to make them suffer. P2

Furthermore, the idea that each individual must lead their own path to recovery was emphasised because of the deeply personal nature of this process. One of the participants reflected on this:

“I believe that everyone has in their mind, their objectives, their wellbeing or where the person feels most comfortable and feels good about oneself, it can be mentally or physically or both together.” P4

This vision emphasised the importance of autonomy and self‐reflection in the search for individual well‐being, recognising that this process can manifest itself in diverse and highly personal ways in each individual.

### 5.2 Stage 2‐Planning

**FIGURE 3:**
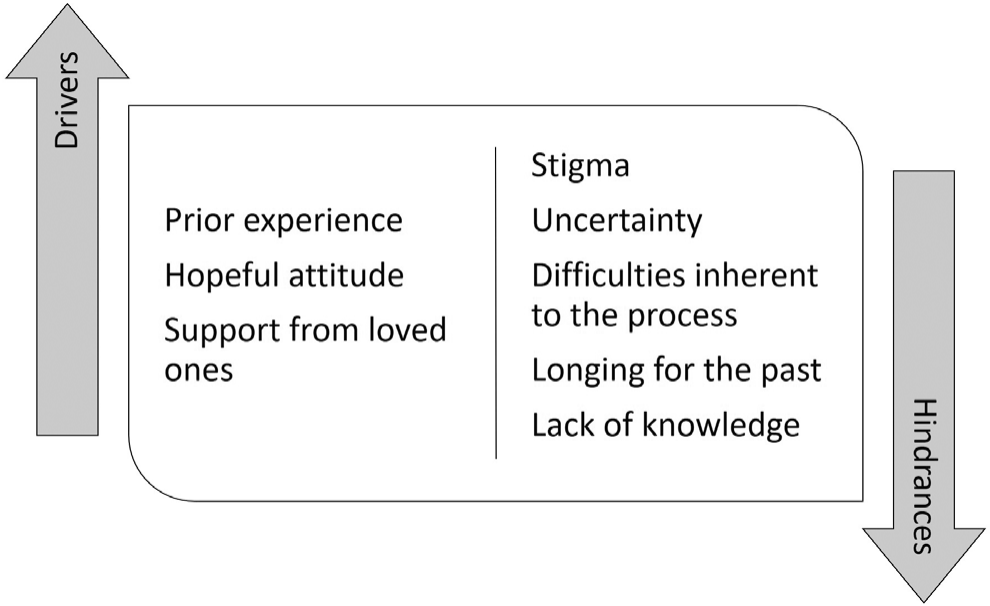
Elements hindering or driving the recovery process from the participants' perspective.

Focus groups were held periodically on a weekly basis. These meetings provided a space for collective discussion in which participants reflected again on the meaning they attributed to the recovery process, as well as elements that hinder or drive the process (Figure 3).

Certain obstacles that hinder the approach to mental disorders were revealed. One of the participants eloquently expressed his vision in this regard:

There are factors that aren't helpful. For example, there is a lot of stigma in society about us and our problems. (P6‐GF3)

This statement strongly underscores the urgency of tackling the stigmas and prejudices associated with mental illness in order to pave the way for the recovery and well‐being of those who suffer from mental illness. The affected person's own lack of knowledge about the nature and evolution of the disorder took centre stage in the groups conducted, as it was related to uncertainty and distressing feelings of not knowing the future…

Sometimes I can't put words to what happens to me because I lack knowledge, we need everyone else to be educated from an early age, and ourselves and our families need to be supported. (P2—GF1)

Another element that emerged as a significant challenge was identified: the uncertainty inherent to the process. Those users who were facing this journey for the first time were immersed in a completely unfamiliar world, full of disconcerting experiences and challenges. This novelty of the process contrasted markedly with the experience of those participants who had already experienced mental health disorders first‐hand. Uncertainty manifested itself as a kind of haze, pervading in every step toward recovery. Newcomers faced a series of dilemmas and new situations that generated doubts and anxieties. Conversely, those with previous experience with mental disorders had a more nuanced perception and deeper understanding of what the process entailed, which gave them a perceptible advantage in coping with it. The gap between novelty and previous experience was revealed as a differentiating factor in the perceived difficulty during the recovery process.

It's not the same when it happens to you the first time, and you don't know what's happening to you, compared to when it happens to you and you know that what's happening to you is normal, that it's happening to other people. (P2‐GF5)

The immense difficulty of the process itself loomed as a major obstacle. Participants recognised that the starkness of confronting their own pre‐mental disorder trajectory, and the intense longing for those aspects of their lives prior to its onset, added an additional layer of complexity to the recovery process. Reliving the memories associated with a stage of life marked by the absence of the disorder, and the feeling of longing for that past reality, acted as stones on the path. The contrast between life prior to the disorder and the current reality was presented as a constant challenge, generating intense emotions and a significant emotional burden that interfere with their process of recovery.

Because you have to do your part, it's very hard, very hard, but you have to try and you have to fight, because it's the only way to do it. (P9‐GF2)

Regarding the factors that help this process, previous experience became relevant in the context of the group discussion, since having been able to experience the partial or absolute recovery from a mental health problem in oneself, means that, in the case of a relapse, the person knows what will come next, the location of the mental health services, the role of each professional…

The first time I fell into depression everything was new, everything was uncertainty. However, when it happens more times, you know that at some point in the future you will be your old self again (P5, GF4)

During this stage, a fundamental factor was identified, that acted as a facilitator in the process: the attitude with which the participants approached their recovery. The importance of facing this path with a hopeful attitude was emphasised, as it was crucial to promote and favour the recovery process. Beyond the mere individual attitude, the relevance of the backing and support from their close circle was emphasised. The personal and emotional support network was identified as a key element facilitating coping with the recovery process, although a lack of such support was recognised as a possible shortcoming for some participants. This emotional and practical support from those closest to them was identified as an essential component in facing the challenges inherent in recovery with greater strength and confidence.

Family and professionals push you to do it, and when you say, ‘I can't do it anymore’, they say ‘no, come on, you can do it.’ (P8, GF2)

If all this had happened to me when I was far away from my parents and friends, I don't know what would have become of me. (P15, GF6)

As a result of these group dynamics, proposals emerged for specific activities that would be implemented in the subsequent phase of the intervention, thus contributing to the development of collaborative nursing care.

### 5.3 Stage 3‐Action

In the third phase of the participatory process, collaboratively created group activities were implemented (Table 2). These activities, designed and planned in the previous phase, were carried out with the participation of users and mental health professionals in a community setting and were implemented following a weekly schedule.

**TABLE 2:** Activities co‐designed collaboratively with participants.

Activity	Short description
Social skills workshops	Practical sessions to improve communication and interpersonal skills.
Cineforum	Screening of a film with a discussion afterwards. Where viewers analyse and discuss the themes. Messages or highlights of the film.
Sexuality education	Workshops on sex education and its particularities for people with mental health problems.
Mindfulness and meditation programmes	Exercises to improve mindfulness and reduce stress
Physical activity	Group or individual exercises adapted to patients' abilities to improve physical and mental well‐being.
Healthy cooking workshop	Learning culinary techniques that promote a balanced and mentally healthy diet
Reading circle	Discussion of books related to self‐improvement. Mental health and well‐being.
problem‐solving workshop	Environments where challenges can be discussed and solutions found together.
Stress management workshops	Techniques and tools for managing stress and anxiety in everyday life.

Among the most outstanding achievements was the creation of a safe and supportive space that facilitated the expression and management of difficult emotions. Most expressed that the sessions allowed them to feel understood and supported by their peers, which was crucial to their recovery process. The participants highlighted that the safe and collaborative environment allowed them to share personal experiences and receive useful advice. Some mentioned that the group activities helped them develop new strategies for managing stress and negative emotions. Group activities promoted greater group cohesion and fostered the development of support networks among participants.

### 5.4 Stage 4‐ Evaluation

At the time of discharge of the users of the mental health day hospital who participated in the intervention, a semi‐structured interview was carried out, which covered the same topics as in Stage 1. This time, we inquired about the perception of collaborative nursing care through the joint creation of group activities and its assessment in the recovery process. Also, during discharge, the quantitative follow‐up assessment was administered to all users in both the intervention and control groups. The quantitative results are shown in Table 3.

**TABLE 3:** Changes in participants' health outcomes after the intervention.

Variable	Control (*n* = 60)		Intervention (*n* = 80)			
	Baseline	Follow‐up	Baseline	Follow‐up	Time	Time × intervention
Recovery Stage 1 Moratorium	26.453 (12.036)	24.188 (10.006)	26.725 (12.211)	26.400 (8.945)	*F* = 2.459 df = 1 *p* = 0.119 *η* = 0.004	*F* = 1.38 df = 1 *p* = 0.242 *η* = 0.002
Recovery Stage 2 Awareness	28.750 (10.374)	29.406 (10.063)	25.863 (10.366)	29.288 (9.621)	*F* = 4.436 df = 1 *p* = 0.037 *η* = 0.010	*F* = 2.042 df = 1 *p* = 0.155 *η* = 0.005
Recovery Stage 3 Preparation	26.813 (10.441)	27.250 (9.879)	23.175 (11.500)	29.512 (9.590)	*F* = 11.947 df = 1 *p* = < 0.001 *η* = 0.025	*F* = 9.060 df = 1 *p* = 0.003 *η* = 0.019
Recovery Stage 4 Rebuilding	26.984 (11.480)	27.844 (10.095)	22.250 (12.075)	29.337 (9.519)	*F* = 17.170 df = 1 *p* = < 0.001 *η* = 0.032	*F* = 10.546 df = 1 *p* = 0.001 *η* = 0.019
Recovery Stage 5 Growth	22.172 (11.020)	23.938 (10.715)	18.250 (11.491)	27.875 (10.966)	*F* = 41.044 df = 1 *p* = < 0.001 *η* = 0.060	*F* = 19.540 df = 1 *p* = < 0.001 *η* = 0.029
WAI‐S total	67.016 (10.293)	68.578 (12.056)	57.112 (13.260)	67.563 (12.043)	*F* = 32.332	*F* = 17.698
					df = 1	df = 1
					*p* = < 0.001	*p* = < 0.001
					*η* = 0.054	*η* = 0.030
Bonds	23.328 (4.543)	23.609 (4.693)	18.700	22.837 (5.117)	*F* = 24.484	*F* = 18.647
			(5.408)		df = 1	df = 1
					*p* = < 0.001	*p* = < 0.001
					*η* = 0.042	*η* = 0.032
Tasks	22.281 (4.177)	22.828 (4.813)	19.238 (5.077)	22.913 (4.866)	*F* = 22.821	*F* = 12.528
					df = 1	df = 1
					*p* = < 0.001	*p* = < 0.001
					*η* = 0.045	*η* = 0.024
Goals	21.406 (3.923)	22.141 (4.276)	19.175 (4.269)	21.813 (4.003)	*F* = 27.065	*F* = 8.622
					df = 1	df = 1
					*p* = < 0.001	*p* = 0.004
					*η* = 0.039	*η* = 0.012
PMH‐Satisfacción Personal	18.875 (6.477)	19.266 (6.373)	17.575	22.650 (6.467)	*F* = 31.456	*F* = 23.106
			(7.277)		df = 1	df = 1
					*p* = < 0.001	*p* = < 0.001
					*η* = 0.039	*η* = 0.029
PMH Prosocial attitude	15.969 (2.624)	15.844 (3.148)	14.488	17.313 (2.504)	*F* = 24.594	*F* = 29.359
			(3.673)		df = 1	df = 1
					*p* = < 0.001	*p* = < 0.001
					*η* = 0.045	*η* = 0.053
PMH‐ Self‐control	11.531 (3.563)	12.000 (4.276)	10.387	13.100 (3.699)	*F* = 31.574	*F* = 15.706
			(3.733)		df = 1	df = 1
					*p* = < 0.001	*p* = < 0.001
					*η* = 0.041	*η* = 0.020
PMH Autonomy	13.391 (3.407)	13.625 (3.675)	12.963	14.000 (3.372)	*F* = 4.249	*F* = 1.694
			(3.644)		df = 1	df = 1
					*p* = 0.041	*p* = 0.195
					*η* = 0.008	*η* = 0.003
PMH‐ Problem‐solving and self‐actualisation	23.813 (5.603)	23.781 (6.497)	21.863	26.188 (5.410)	*F* = 23.871	*F* = 24.570
			(4.414)		df = 1	df = 1
					*p* = < 0.001	*p* = < 0.001
					*η* = 0.036	*η* = 0.037
PMH Interpersonal relationship skills	17.906 (4.569)	18.344 (4.782)	18.038	22.300 (4.591)	*F* = 48.805 df = 1	*F* = 32.324
			(4.465)		*p* = < 0.001	df = 1
					*η* = 0.057	*p* = < 0.001
						*η* = 0.037
PMH total	101.484 (17.064)	102.859 (21.378)	95.313 (20.155)	115.550 (19.201)	*F* = 50.853	*F* = 38.735
					df = 1	df = 1
					*p* = < 0.001	*p* = < 0.001
					*η* = 0.067	*η* = 0.051

Abbreviations: PMH, positive mental health; WAI‐S, working alliance inventory‐short.

Regarding the STORI, in the “Moratorium” phase of recovery, there were no significant differences before and after the intervention. In the “Awareness” phase, differences are observed before and after the intervention; however, there are no differences by group (intervention/control). In the “Preparation”, “Rebuilding” and “Growth” phases, however, statistically significant differences can be observed between before and after and in the intervention groups with a small and medium effect size in some cases. Thus, we can observe that the scores of the intervention group improve more than those of the control group.

Regarding the total TR, statistically significant differences are observed before and after the intervention with a medium effect size and also by group, with a small effect size. Thus, the scores of the intervention group improve more than those of the control group.

Concerning the factors of the TR, there were statistically significant differences before and after the intervention and also by group in bonding, tasks and objectives, in all cases with a small effect size. Therefore, the intervention group scores improved more than those of the control group.

In the case of PMH, there were statistically significant differences before and after the intervention and also by group in the six phases, in all cases with a small effect size, with the exception of “Autonomy”, where there were no differences by group. Thus, the intervention group scores improved more than those of the control group. Finally, regarding the total PMH, statistically significant differences were found before and after the intervention and also between the two groups, with a mean effect size. The scores improved in both cases, with a greater improvement in the intervention group.

Integrating the qualitative and quantitative results in this assessment phase, in terms of the changes produced in the recovery process, the participants highlighted that the co‐creation of group activities allowed them to feel more involved. Most felt that the collaborative group activities were an essential component in their recovery process. Many expressed that this active participation helped them develop a sense of belonging and purpose, which was crucial to their emotional well‐being. In fact, the quantitative results indicate that the intervention had a significant impact for those users who scored higher in the later stages of the recovery process. In contrast, the intervention did not show significant changes for those who were in the early stages.

Regarding the level of PMH, users who received the intervention particularly valued the opportunity to share their experiences and learn from others, which strengthened support networks and fostered an atmosphere of empathy and mutual understanding. Participants mentioned that the group activities not only improved their emotional well‐being, but also provided them with practical tools to manage stress and daily difficulties. These findings are confirmed from the quantitative perspective, as the intervention showed a significant impact on improving the overall PMH levels of the users who received the collaborative nursing care, increasing personal satisfaction, prosocial attitude, self‐control and problem‐solving. In this regard, participants noted that, thanks to the group activities, they felt more prepared to face life outside the day hospital. The collaboration and mutual support experienced during the sessions were identified as key factors contributing to their progress. These findings also coincide with the increase in the levels of interpersonal skills related to the PMH of the users in the intervention group. Indeed, the quantitative results also indicate that in the intervention group, the TR was significantly improved compared to the control group. Specifically, positive changes were observed in several stages of the recovery process and in PMH factors.

## 6 Discussion

The aim of this study was twofold: to explore the process of change produced in the recovery process in mental health day hospital users who received the nursing collaborative care intervention and, secondly, to evaluate the impact of the collaborative care intervention. The results obtained show that the collaborative nursing care intervention in mental health day hospitals had a positive quantitative impact on various areas of the recovery process and patients' PMH. Likewise, the intervention improved the quality of the TR with the nurse. Also, our qualitative findings showed that users perceived that collaborative group activities were an essential component in their recovery process since they helped them develop a sense of belonging and purpose, strengthening support networks and fostering an environment of empathy and mutual understanding, crucial aspects also for a better PMH.

Regarding the results obtained concerning the stage of the recovery process, the significant improvements observed in the preparation, reconstruction and growth stages of the recovery process suggest that collaborative nursing care facilitates a more effective and sustained recovery. However, the lack of significant change at the moratorium stage indicates that the intervention may not have an immediate impact in the early stages of recovery, possibly due to the severity of the patients' initial condition. This suggests that there is likely a need for different types of interventions specifically targeted at individuals in the early stages. No evidence has been found indicating the effectiveness of interventions differentiated by stage of recovery. However, the progress observed in later stages highlights the importance of mental health nurses in maintaining a continuous and collaborative approach to their interventions. These results are consistent with the literature which emphasises the need for sustained and personalised interventions to achieve meaningful recovery in patients with severe mental disorders (Hornik‐Lurie et al. 2018; Tamayo and Lane 2022; Winsper et al. 2020).

Relevant findings are significant increases in PMH factors such as personal satisfaction, prosocial attitude, self‐control and interpersonal relationship skills. These results indicate that collaborative care interventions not only address symptoms of mental disorders, but also promote patients' general well‐being and social functioning. The limited improvement in autonomy suggests that, although collaborative care is effective in many ways, nurses should be aware that additional interventions or different approaches may be needed to strengthen patients' autonomy.

An important aspect to highlight from the results for nursing practice is the improvement in the TR, as measured through the components of bonds, tasks and goals, suggesting that collaborative care strengthens the connection between patients and nurses. This strengthening is crucial, as a strong TR is associated with better outcomes in the recovery of people with mental health problems. The intervention appears to have improved the patients' ability to engage with the therapeutic process and work toward their goals, which is consistent with the literature that stresses the importance of a positive TR for treatment success (Coelho et al. 2024; Marchi et al. 2024).

The results of this study are in line with previous research that has highlighted the benefits of collaborative approaches to mental healthcare (Boerkoel and Brommels 2022; Sowers 2012). Previous studies have shown that active involvement of patients in their own care and collaboration between healthcare professionals leads to better clinical outcomes and increased patient satisfaction. This study contributes to the growing evidence that collaborative care can be an effective strategy for mental health nurses to improve both clinical outcomes and the overall well‐being of patients with mental health problems.

### 6.1 Strengths and Limitations

The sample used in the study was relatively small and limited to the mental health day hospital setting, which may affect the generalisation of the results to other populations and settings. A greater diversity in the sample might have provided a more complete picture of the effects of collaborative nursing care in different settings. Study participants presented with a variety of mental health diagnoses. Although this reflects the diversity of a real clinical population, it also introduces variability that may complicate interpretation of the results. Non‐probability sampling was used, meaning that participants were not randomly selected. In addition, it should be noted that the baseline results for some variables differed between the control group and the intervention group. The team found no explanation for these differences. Random probability sampling would have increased the representativeness of the sample and improved the generalisability of the findings to the general population of people with mental health problems.

Concerning strengths, the study was designed with a longitudinal analysis that enabled the assessment of the effects of collaborative nursing care at multiple points over time. This provided a dynamic perspective of how patients perceive and benefit from these interventions at different stages of their recovery process.

## 7 Conclusions

This study provides evidence that collaborative nursing care in mental health day hospitals significantly improves the recovery process, TR and various aspects of users' PMH. Patients reported greater personal satisfaction, a more positive prosocial attitude, improved self‐control, greater autonomy and improved problem‐solving and interpersonal relationship skills. These findings suggest that collaborative nursing care not only improves clinical outcomes, but also contributes to greater patient well‐being and quality of life. Qualitative interviews corroborated these quantitative findings, highlighting the patients' positive perception of collaboration in their recovery process and the importance of feeling empowered and listened to. The therapeutic relationship emerged as a central factor in recovery, facilitating an environment of trust and support that is essential for patients' emotional and psychological well‐being.

## 8 Implications to Clinical Practice

The results of this study have important implications for both nursing practice and mental health nursing research. The evidence obtained underscores the effectiveness of collaborative and participatory approaches by mental health nurses for patient recovery, suggesting that the active involvement of clients in the creation of group activities is beneficial. Day hospitals and other treatment settings should consider implementing collaborative nursing care programs to improve patient outcomes.

Future research should further explore the specific mechanisms through which collaborative nursing care impacts mental health and evaluate the long‐term effectiveness of these approaches. Also, studies could investigate ways to optimise group activities for different populations and contexts, as well as to determine differences by gender.

## 9 Relevance Statement

This study is highly relevant to mental health nursing practice, providing evidence‐based insights into the effectiveness of collaborative nursing care in improving patient outcomes in mental health day hospitals. By focusing on the nurse–patient relationship and the role of nurses in facilitating mental health recovery, the research highlights the critical contribution of nursing practices to holistic patient care. The findings have the potential to influence clinical practice, making collaborative nursing care an essential tool in enhancing the recovery process, positive mental health and the therapeutic relationship.

## 10 Reporting Checklist

The study adhered to CONSORT and COREQ guidelines.

## Ethics Statement

Authorisation has been obtained from the management of the centres and approval for the project has been granted by the Ethics Committee of the institution where the study will be carried out, FIDMAG Hermanas Hospitalarias (PR‐2020‐10).

## Conflicts of Interest

The authors declare no conflicts of interest.

## Data Availability

The data that support the findings of this study are available from the corresponding author upon reasonable request.
